# The Effects of Green Spaces and Noise Exposure on the Risk of Ischemic Stroke: A Case–Control Study in Lebanon

**DOI:** 10.3390/ijerph21101382

**Published:** 2024-10-19

**Authors:** Jad El Masri, Hani Finge, Ahmad Afyouni, Tarek Baroud, Najla Ajaj, Maya Ghazi, Diala El Masri, Mahmoud Younes, Pascale Salameh, Hassan Hosseini

**Affiliations:** 1 INSERM U955-E01, Institut Mondor de Recherche Biomédicale, Université Paris-Est Créteil, 94010 Créteil, France; consultation.hosseini@gmail.com; 2 École Doctorale Sciences de la Vie et de la Santé, Université Paris-Est Créteil, 94010 Créteil, France; 3 Faculty of Medical Sciences, Lebanese University, Beirut 1533, Lebanonpascalesalameh1@hotmail.com (P.S.); 4 INSPECT-LB (Institut National de Sant e Publique, d’Épidemiologie Clinique et de Toxicologie-Liban), Beirut 1103, Lebanon; 5 Department of Neurology, Faculty of Medical Sciences, Lebanese University, Beirut 1533, Lebanon; 6 School of Medicine, Lebanese American University, Byblos 1102, Lebanon; 7 Faculty of Medicine, University of Balamand, Koura 1100, Lebanon; 8 Faculty of Pharmacy, Lebanese University, Beirut 1533, Lebanon; 9 Department of Primary Care and Population Health, University of Nicosia Medical School, 2417 Nicosia, Cyprus; 10 Department of Neurology, Henri Mondor Hospital, AP-HP, 94010 Créteil, France

**Keywords:** ischemic stroke, risk factors, environment, green space, noise exposure

## Abstract

Background: Environmental surroundings reduce the rate of several diseases, especially those related to stressful events. Ischemic stroke can be affected by such events, either directly or through its risk factors. Therefore, the present study evaluates the effects of green spaces and noise exposure on the risk of ischemic stroke. Methods: A case–control study was carried out, including 200 ischemic stroke cases within the first 48 h of diagnosis and 200 controls, divided equally into hospitalized and non-hospitalized participants. Controls were matched to cases based on age and gender. Socio-demographic characteristics were assessed, in addition to environmental surroundings and noise exposure at home and at workplaces. Results: Living in a house, having a house garden, and taking care of the garden were associated with a lower risk of suffering an ischemic stroke (*p* < 0.001, *p* < 0.001, and *p* = 0.009, respectively). However, having buildings as the view from home led to a higher stroke rate (*p* < 0.001). Working in an urban area, the workplace being surrounded by buildings, and the workplace not being surrounded by green spaces were also associated with a higher risk of suffering an ischemic stroke (*p* = 0.002, *p* = 0.001, and *p* = 0.03, respectively). As for noise exposure, being exposed to traffic noise, human noise, and other types of noise was significantly associated with a higher risk of ischemic stroke, while being exposed to higher levels of natural noise was significantly associated with a lower risk of ischemic stroke. Higher levels of noise were also associated with higher risks of ischemic stroke in homes and workplaces (*p* < 0.001 and *p* = 0.008, respectively). Conclusions: Environmental surroundings and noise exposure were found to affect the risk of ischemic stroke. Greater green spaces and lower noise exposure play a protective role against ischemic stroke, suggesting a possible prevention strategy through environmental modifications at home and workplaces.

## 1. Background

With the huge rise in its incidence, ischemic stroke remains one of the major causes of mortality and disability worldwide [1]. Stroke has a great impact on the quality of life of patients and their families, especially those who provide long-term, day-to-day care [2]. Professional attention and support are necessary to maintain physical and emotional health and to decrease the stroke burden [3].

Risk factors for stroke can be categorized as modifiable and nonmodifiable. Age, sex, and race are nonmodifiable, while hypertension, smoking, diet, and physical inactivity are the main reported modifiable risk factors, making up around 90% of all stroke-related risk factors [4]. Another risk factor for stroke is the residential environment. For instance, exposure to air pollution was found to increase the risk of stroke by resulting in vascular dysfunction following the formation of plaques and the development of oxidative stress [5,6].

Globally, a dramatic demographic shift towards urbanization is occurring. Whilst it clearly has health impacts due to the limited green space environments, there is uncertainty as to whether the purported health benefits of green spaces are an urban myth or fact. Urbanization is expanding, and hence green spaces have been fragmented in many regions, leading to the degradation of the environment, which consequently causes major health problems [7]. In the last few decades, various studies have highlighted the importance of green space areas on health outcomes, including pregnancy, mental health, and, especially, cardiovascular diseases [8]. The pathophysiological mechanism for this association is unclear. However, physical and psychological benefits have been linked to green spaces through their purported effects on physical activity [9]. Numerous health benefits of physical activity have been documented, such as the effects on cardio- and cerebro-vascular disease, diabetes, colorectal cancer, osteoporosis, depression, and fall-related injuries [10,11,12]. It also improves mental functioning, mental health, and well-being [13] and may have long-lasting psychological benefits [14].

Recently, it has been suggested that green space exposure may influence the risk of CVD by reducing air pollution and relieving stress [15]. More precisely, some studies reported a correlation between CVD and green spaces: an increase in green space exposure was linked to decreased hospitalization with CVDs [15]. For instance, a study conducted in Perth showed a lower hospitalization rate among people living in the highest tertile of green spaces compared to those living in the lowest [16]. Despite the strong association between green space exposure and vascular health, there are no clear data on this relationship yet [17].

Despite not being an industrial country, the level of pollution in Lebanon has reached critical levels [18,19,20]. Diesel generators, along with heavy traffic, are the main contributors to air pollution, besides increasing noise levels [21,22]. Furthermore, the development of real estate and the unorganized city development have reduced the availability of natural environments.

Considering this evidence, and given the context, this study aims to assess the effects of green spaces and noise exposure in residential and workplaces on ischemic stroke risk. 

## 2. Methods

### 2.1. Study Design

A case–control study was carried out to assess the effects of green spaces and noise exposure on ischemic stroke. All participants were informed about the study’s details and aims, and all participants were assured that the data collected were confidential. Participation was optional, and data were collected between March 2023 and December 2023 from patients’ medical records in addition to face-to-face interviews.

### 2.2. Participants

All participants were Lebanese, aged 18 or above, and admitted after having an ischemic stroke to Sahel General Hospitals or Al Rassoul Al Azam Hospital in Beirut.

Inclusion criteria: To be included, a patient needs to be within the first three days of the observational period following the incidence of ischemic stroke, which was diagnosed using computed tomography (CT) and/or magnetic resonance imaging (MRI) besides clinical confirmation [23]. As for controls, gender- and age-matching (with a 3-year range) were needed, in addition to the absence of stroke, stroke history, or stroke risk factors such as hypercoagulability and cerebrovascular diseases. Controls were recruited from patients in the same hospitals (48%), including outpatients, and from the general population (52%).

Exclusion criteria: All cases with no consent were excluded, in addition to cases with no clinical and radiological confirmation of ischemic stroke. Also, other types of cerebrovascular attack (CVA) were excluded, such as transient ischemic attack or hemorrhagic stroke [24]. As for controls, exclusion was based on the lack of consent and the admission of vascular-related issues.

### 2.3. Variables and Data Source Measures

The questionnaire was filled using patient’s medical file records and by a 10 min face-to-face interview.

Socio-demographic characteristics of each participant were assessed, such as age, gender, marital status, education, and employment. Disability level was also assessed for cases using Modified Rankin Scale, classifying each patient’s disability level from 0 (no disability) to 6 (death) [25].

As for the environmental surroundings, each participant was asked about the place of residency, the place of work, and roads in between, in addition to their surroundings and the presence of private garden.

Regarding noise exposure, a Likert scale was used to assess how loud it is at home- and workplace, in addition to the level of exposure to traffic noise, human noise, other noise, and natural sound.

### 2.4. Ethical Considerations

This study respects confidentiality and anonymity. Ethical approval was granted from the Institutional Review Board (IRB) at the hospitals included in data collection (ID number: 1/2023).

### 2.5. Statistical Analysis

Data were analyzed using SPSS software version 25. Descriptive analysis was performed using frequencies and percentages (categorical variables) and means and standard deviations (continuous variables). Bivariate analysis was performed to identify potential risk of environmental surroundings on ischemic stroke, including green spaces and noise exposure. Student’s test was utilized to compare means between two groups, and ANOVA test was utilized to compare means between more than two groups. The Chi-square and Fisher exact tests were used to compare percentages between two groups. *p* < 0.05 was considered statistically significant.

A stepwise forward binomial logistic regression model was performed to investigate the odds ratio (OR) with a 95% CI of marital status; educational level; living location and its surrounding; work location; home exposure to traffic, noise, humans, and natural sounds; and loudness around home among participants with ischemic stroke and the control group. Hosmer–Lemeshow test was non-significant, demonstrating the test’s adequacy. All covariates with a *p* < 0.2 in the bivariate analysis were included in the logistic regression model. The CI was set at 95%, and a value of *p* < 0.05 was considered significant.

## 3. Results

### 3.1. Effect of Socio-Demographic Factors on Ischemic Stroke

Table 1 shows the socio-demographic characteristics of cases and controls in this study, where a total of 400 participants, divided into 200 cases and 200 controls, were included. The mean age of cases was 68.34 ± 13.267, and that of controls was 66.42 ± 14.575; *p* = 0.169. Males were predominant in both groups, having a percentage of 52 in the cases and 50.5 in controls (*p* = 0.772). There was a significant difference in marital status and education between cases and controls (*p*-value < 0.001), while no significance was reported in employment (*p*-value = 0.65).

### 3.2. Stroke-Related Characteristics in Ischemic Stroke Patients

Table 2 shows the disability level, number of strokes, and age at first stroke for the cases included. Around one-third of cases (63 cases; 31.5%) had moderate disability (mRS score = 3), around a quarter (47 cases; 23.5%) had slight disability (mRS score = 2), and around one-fifth (43 cases; 21.5%) had moderate–severe disability (mRS score = 4). Only one case (0.5%) had no clinical symptoms, and eight cases (4%) died. The majority of patients enrolled had only one stroke (139 participants, 69.5%), a quarter had two strokes (24.5%), and the average age at the first stroke was 67.435 ± 13.087.

### 3.3. The Correlation Between Loudness, Residential Characteristics, and Green Space Exposure

Table 3 shows the association between loudness, residential characteristics, and green space exposure. Living in a house, having a personal garden, and taking care of it were significantly associated with a higher exposure to loudness around the house (*p* < 0.001 each). Having a home surrounded by buildings, and not by green spaces, was also significantly associated with higher noise exposure (*p* < 0.001 each).

As for the workplace, those having a job and being surrounded by buildings and not green spaces were significantly associated with a higher level of loudness (*p* < 0.001 each).

### 3.4. Effects of Residential Characteristics and Green Space Exposure on Ischemic Stroke

Table 4 shows the association between residential characteristics and green space exposure in cases and controls.

The number of stroke patients living in a house is significantly lower than controls (35.02% vs. 76.93%, *p* < 0.001), as is the number of cases with a house garden compared to controls (15% vs. 42%, *p* < 0.001). Moreover, the number of cases having buildings as a view from their home (196, 98%) is significantly higher than controls (151, 75.5%) (*p*-value < 0.001).

The number of stroke cases working in rural areas (8, 4%) is significantly lower than controls (27, 13.5%) (*p*-value = 0.002). Moreover, the number of stroke cases with a view of buildings from their workplace is significantly higher than controls (91.23% vs. 66.67%, *p* = 0.001), while the number of cases with a view of green space is significantly lower than controls (29.82% vs. 49.2%, *p* = 0.03). In addition, all stroke cases have buildings as a view on the road to their work (57, 100%), which is significantly higher than controls (44, 69.84%) (*p*-value < 0.001).

### 3.5. Effects of Noise Exposure on Ischemic Stroke

Table 5 shows the association between noise exposure and ischemic stroke in cases and controls.

Regarding the noise from the home, there was a significant difference in exposure to noise between cases of ischemic stroke and controls (*p*-value < 0.001 in every noise type and natural sounds), with cases of ischemic stroke being more exposed to noise. Specifically, almost two-thirds (61%) of cases were exposed to moderate or higher traffic noise compared to 37.5% of controls, 59.5% of cases to “other” noises compared to 28% of controls, 78.5% of cases to human sounds compared to 68% of controls, and 76% of cases reported loud surroundings compared to 38% of controls. Conversely, 55.5% of controls were exposed to natural sounds at moderate or higher levels, compared to 40% of cases.

Similarly, regarding noise from the workplace, there was a significant difference in exposure to noise between cases of ischemic stroke and controls (with a *p*-value < 0.05 in every noise type and natural sounds except traffic noise), with cases of ischemic stroke being more exposed to noise. Nearly two-thirds of cases (64.9%) were exposed to moderate or higher levels of “other” noises compared to 50.78% of controls, and 85.95% of cases were exposed to moderate or higher levels of human sounds compared to 58.72% of controls. Additionally, 85.94% of cases reported loud surroundings in a moderate or higher category compared to 55.54% of controls. Conversely, almost a quarter of controls (26.97%) were exposed to natural sounds at moderate or higher levels, compared to just 8.76% of cases.

Table 6 shows that the level of loudness around the house was not correlated to the level of disability on admission. However, the level of loudness around the workplace had a significant correlation with disability level (*p* = 0.028), where the only patient not being exposed to noise at all had a low level of disability, and around half of those slightly exposed had low (42.85%) or moderate (42.85%) disability levels, while the majority of those exposed to extreme noise (66.66%) had severe disability levels.

Table 7 shows the forward stepwise binomial regression of the incidence of ischemic stroke: being single or married versus widowed and having had healthcare education were associated with lower odds of ischemic stroke, while living in a building, having a home surrounded by other buildings, and exposure to many types of noise (traffic and industrial) were associated with higher odds of ischemic stroke.

## 4. Discussion

In this case–control study, we explored the relationship between exposure to green spaces, noise, and the incidence of stroke in the Lebanese population. The results showed a significant association between diminished exposure to green spaces, higher exposure to urban environments (e.g., buildings and traffic), and increased noise levels at both home and work with the incidence of ischemic stroke.

This study contributes to the growing body of evidence linking green spaces and noise exposure to cardiovascular health, particularly stroke risk [26]. Our findings corroborate the protective role of green spaces, which have been shown to positively impact multiple health outcomes. Numerous studies have suggested that increased exposure to greenness is beneficial for decreasing the risk of childhood asthma [27], Parkinson’s disease [28], Alzheimer’s disease [29], inflammatory bowel disease [30], and cancer [31], in addition to mortality and cardiovascular risk factors [32,33]. However, noise exposure has been implicated to negatively impact different diseases such as diabetes [34], atrial fibrillation [35], and ischemic heart disease [36].

There are several possible explanations for how increased greenness and decreased noise can positively impact stroke risk. Two of the main possibilities are described hereafter. To begin with, access to green spaces is closely related to physical activity levels, which in turn influences cardiovascular health. Colom et al. highlighted that access to public open spaces significantly correlates with increased physical activity, particularly in high-risk populations and older adults specifically [37,38]. In fact, the presence of green spaces is believed to encourage healthier lifestyle behaviors, such as walking and social interaction, which can reduce the likelihood of obesity, type 2 diabetes, and stroke [39,40,41]. In Lebanon, physical activity is considered under level, especially in older ages [42]. Hence, increased green space exposure is a necessity, as it encourages physical activity in older adults, which in turn can decrease the risk of stroke in this particular high-risk population.

Second, good mental health is known to decrease the risk of ischemic stroke [43]. For instance, diagnoses with depression or other mental health-related disorders, in addition to psychiatric hospitalization, were found to increase stroke rates [44,45]. Moreover, studies have shown that mental health outcomes have been associated positively with green space and negatively with noise exposure [46,47,48]. Therefore, increasing green spaces and decreasing noise exposure enhance mental health, which in turn can decrease the risk of ischemic stroke. In Lebanon, the high levels of psychological distress highlight the need for such measures [49].

The results of this study, alongside the findings from recent research on street-level and residential green spaces [50,51], highlight the urgent need for public health policies that promote access to green spaces and reduce noise pollution, especially in urban areas. Urban planning should consider integrating street-level vegetation, not just large parks, into residential and workplace environments. In addition, Ramos-Lima, M. J. M. et al.’s findings showed an inversely proportional relationship between the severity of stroke and quality of life [52]. Moreover, quality of life is an important aspect of health that is enhanced by the availability of parks and green areas [53] and decreased with noise exposure [54]. Another point worth mentioning is the correlation between greenness and disability levels. Similar to our findings, a study by Zhu, A. et al. showed that increased exposure to green spaces reduced disability levels [55]. This highlights the importance of increased green spaces and decreased noise exposure, which increases the quality of life and reduces disability levels in stroke patients.

This study has some limitations worth mentioning. Due to the case–control nature of this study, a potential for recall bias is present, where participants may have recalled their characteristics with a lack of precision. This was especially particular after a stroke, where a family member interfered in some cases. Moreover, the matching between cases and controls was based on age and gender, disregarding other factors. For instance, the educational level was significantly different between cases and controls, which might affect the credibility of self-reported information. Furthermore, other factors, such as air pollution, physical activity, and mental health, are important factors that can be directly related to the study objective yet were not assessed. Another factor that was missing was the functional outcomes, as this study lacked follow-up. Future research should continue to explore the mechanisms underlying the relationship between green spaces and stroke, such as assessing direct relations to physical activity and mental health. Longitudinal studies would further clarify the temporal relationship between environmental exposures and stroke risk. Also, prospective studies are needed to assess functional outcomes and correlate them to greenness and noise exposure.

## 5. Conclusions

In conclusion, this study adds to the evidence that green spaces and noise exposure positively impact health outcomes in a developing country, particularly stroke risk. This findings align with previous research highlighting the importance of green spaces in promoting physical activity, mental health, metabolic health, and quality of life. Urban planners and policymakers should prioritize creating and maintaining accessible green spaces and calm areas in urban environments to promote cardiovascular health and well-being across populations, particularly as the global population ages and cities become more densely populated.

## Figures and Tables

**Table 1:** Bivariate analysis of socio-demographic characteristics associated with ischemic stroke.

Factor	Category	Cases		Control		*p*-Value
		Number	Percentage	Number	Percentage	
Gender	Female	96	48	99	49.5	0.772
	Male	104	52	101	50.5	
Marital Status	Single	13	6.5	19	9.5	<0.001 *
	Married	113	56.5	139	69.5	
	Divorced	4	2	4	2	
	Widowed	70	35	38	19	
Education	Not educated	50	25	81	40.5	<0.001 *
	School education	93	46.5	71	35.5	
	Non-healthcare education	53	26.5	31	15.5	
	Health care education	4	2	17	8.5	
Employment	Not employed	143	71.5	136	68	0.65
	Employed	26	13	28	14	
	Free profession	31	15.5	36	18	
Age	Mean + SD	68.34 ± 13.267		66.42 ± 14.575		0.169

* Represents *p* < 0.05.

**Table 2:** Descriptive analysis of stroke-related characteristics in ischemic stroke patients.

Factor	Category	Number	Percentage
mRS score	0	1	0.50
	1	6	3.00
	2	47	23.50
	3	63	31.50
	4	43	21.50
	5	32	16.00
	6	8	4.00
Number of strokes	1	139	69.50
	2	49	24.50
	3	10	5.00
	4	2	1.00
Age	Mean ± SD	67.380 ± 13.952	
Age at 1st stroke	Mean ± SD	67.435 ± 13.087	

**Table 3:** Bivariate analysis of the association between residential characteristics and noise exposure.

Factor	Category	Level of Loudness					*p*-Value
		Not At All	Slight	Moderate	Very	Extreme	
How loud around house							
Live in	Building	9	54	97	71	26	<0.001 *
	House	18	91	24	8	2	
If building, which floor	Mean ± SD	3.44 ± 2.651	2.68 ± 1.669	3.68 ± 1.955	3.67 ± 2.126	2.26 ± 1.710	0.014 *
House Garden	Yes	16	70	22	3	1	<0.001 *
	No	11	75	99	76	27	
Care of garden	Yes	13	53	14	0	2	<0.001 *
	No	14	92	107	79	26	
View from your home							
Buildings	Yes	18	116	109	77	27	<0.001 *
	No	9	29	12	2	1	
Green spaces	Yes	24	104	65	5	1	<0.001 *
	No	3	41	56	74	27	
How loud around workplace							
Work in	Rural area	2	13	14	4	2	0.066
	Urban area	5	16	27	27	10	
Work per month (hours)	Mean ± SD	39.14 ± 12.047	38.07 ± 20.933	49.14 ± 14.068	46.06 ± 18.766	45.25 ± 18.621	0.088
View from your workplace							
Buildings	Yes	3	15	36	28	12	<0.001 *
	No	4	14	5	3	0	
Green spaces	Yes	5	21	17	4	1	<0.001 *
	No	2	8	24	27	11	

* Statistically significant.

**Table 4:** Bivariate analysis of the association between residential characteristics and green space exposure and ischemic stroke.

Factor	Category	Cases		Control		*p*-Value
		Number	Percentage	Number	Percentage	
Home						
Live in	Building	167	64.98	33	23.07	<0.001 *
	House	90	35.02	110	76.93	
If building, which floor	Mean ± SD	3.467 ± 1.919		3.044 ± 2.156		0.122
House Garden	Yes	30	15	82	41	<0.001 *
	No	170	85	118	59	
Care of garden	Yes	17	20.73	65	79.27	0.009 *
	No	65	79.27	17	20.73	
View from your home						
Buildings	Yes	196	98	151	75.5	<0.001 *
	No	4	2	49	24.5	
Green spaces	Yes	98	49	101	50.5	0.764
	No	102	51	99	49.5	
Workplace						
Work in	Rural area	8	4	27	13.5	0.002 *
	Urban area	49	24.5	37	18.5	
	Do not work	143	71.5	136	68	
Work per month (hours)	Mean ± SD	47.368 ± 13.699		42.317 ± 20.504		0.113
View from your workplace						
Buildings	Yes	52	91.23	42	66.67	0.001*
	No	5	8.77	21	33.33	
Green spaces	Yes	17	29.82	31	49.2	0.03 *
	No	40	70.18	32	50.8	
Road to Workplace						
Time to workplace (minutes)	Mean ± SD	23.772 ± 12.147		27.889 ± 31.908		0.362
Stuck in traffic	Yes	20	35.09	19	28.79	0.454
	No	37	64.91	47	701.21	
Time in traffic (minutes)	Mean ± SD	20.75 ± 9.497		23.333 ± 18.663		0.583
View on the road to your workplace						
Buildings	Yes	57	100	44	69.84	<0.001 *
	No	0	0	19	30.16	
Green spaces	Yes	31	56.36	29	46.77	0.30
	No	24	43.64	33	53.23	

* Represents *p* < 0.05.

**Table 5:** Bivariate analysis of the association between noise exposure and ischemic stroke.

Factor	Category	Cases		Control		*p*-Value
		Number	Percentage	Number	Percentage	
Noise from your home						
Traffic noise (cars, buses, airplanes, motorcycles)	Not at all	6	3.00	47	23.50	<0.001 *
	Slight	72	36.00	78	39.00	
	Moderate	65	32.50	29	14.50	
	Very	43	21.50	27	13.50	
	Extreme	14	7.00	19	9.50	
Other noises (sirens, constructions, industry, loading of goods…)	Not at all	10	5.00	82	41.00	<0.001 *
	Slight	71	35.50	62	31.00	
	Moderate	69	34.50	31	15.50	
	Very	38	19.00	15	7.50	
	Extreme	12	6.00	10	5.00	
Sounds of human beings (conversation, laughter, children at play, footsteps…)	Not at all	1	0.50	28	14.00	<0.001 *
	Slight	42	21.00	62	31.00	
	Moderate	85	42.50	67	33.50	
	Very	55	27.50	26	26.00	
	Extreme	17	8.50	17	8.50	
Natural sounds (singing birds, flowing water, wind in vegetation…)	Not at all	55	27.50	38	14.00	<0.001 *
	Slight	65	32.50	51	25.50	
	Moderate	50	25.00	52	26.00	
	Very	29	14.5	37	18.50	
	Extreme	1	0.50	22	11.00	
How loud the noise is around your house	Not at all	5	2.50	22	11.00	<0.001 *
	Slight	43	21.5	102	51.00	
	Moderate	73	36.5	48	24.00	
	Very	60	30.00	19	9.50	
	Extreme	19	9.50	9	4.50	
Noise from your workplace						
Traffic noise (cars, buses, airplanes, motorcycles)	Not at all	3	52.63	11	17.46	0.188
	Slight	16	28.08	21	33.33	
	Moderate	21	36.84	16	25.39	
	Very	12	21.05	9	14.28	
	Extreme	5	8.77	6	9.52	
Other noises (sirens, constructions, industry, loading of goods…)	Not at all	3	52.63	15	23.80	0.046 *
	Slight	17	29.82	16	25.39	
	Moderate	17	29.82	18	28.57	
	Very	14	24.56	9	14.28	
	Extreme	6	10.52	5	7.93	
Sounds of human beings (conversation, laughter, children at play, footsteps…)	Not at all	1	1.75	8	12.69	0.004 *
	Slight	7	12.28	18	28.57	
	Moderate	24	42.10	18	28.57	
	Very	21	36.84	11	17.46	
	Extreme	4	7.01	8	12.69	
Natural sounds (singing birds, flowing water, wind in vegetation…)	Not at all	36	63.15	24	38.09	0.04 *
	Slight	16	28.07	22	34.92	
	Moderate	3	5.26	7	11.11	
	Very	1	1.75	6	9.52	
	Extreme	1	1.75	4	6.34	
How loud the noise is around your workplace	Not at all	1	1.75	6	9.52	0.008 *
	Slight	7	12.28	22	34.92	
	Moderate	25	43.85	16	25.39	
	Very	18	31.57	13	20.63	
	Extreme	6	10.52	6	9.52	

* Represents *p* < 0.05.

**Table 6:** Bivariate analysis of the level of loudness around home and workplace associated with disability level (mRS) in ischemic stroke.

Category	Factor	mRS Categories			Total	*p*-Value
		0 Till 2	3 Till 4	5 Till 6		
How loud it is around your house	Not at all	1 (20%)	1 (20%)	3 (60%)	5	0.322
	Slight	13 (30.23%)	21 (48.83%)	9 (20.93%)	43	
	Moderate	17 (2.28%)	42 (57.53%)	14 (19.17%)	73	
	Very	20 (33.33%)	29 (48.33%)	11 (18.33%)	60	
	Extreme	3 (15.78%)	13 (68.42%)	3 (15.78%)	19	
How loud it is around your house	Not at all	1 (100.00%)	0 (0.00%)	0 (0.00%)	1	0.028 *
	Slight	3 (42.85%)	3 (42.85%)	1 (14.28%)	7	
	Moderate	13 (52.00%)	10 (40%)	2 (8.00%)	25	
	Very	8 (44.44%)	9 (50.00%)	1 (5.55%)	18	
	Extreme	1 (16.66%)	1 (16.66%)	4 (66.66%)	6	

* Statistically significant.

**Table 7:** Multivariable analysis: stepwise forward binomial regression of the incidence of ischemic stroke.

Independent Variables	*p*-Value	OR	CI 95%	
			Lower Bound	Upper Bound
Marital Status † Widowed	0.007 *			
Single	0.003 *	0.177	0.057	0.551
Married	0.002 *	0.319	0.154	0.663
Divorced	0.251	0.334	0.051	2.177
Educational Level † Healthcare-related	<0.001 *			
Not educated	0.356	1.955	0.471	8.105
School education	0.005 *	7.162	1.789	28.667
Non-healthcare related education	0.009 *	6.901	1.631	29.204
Live in a building † House	0.008 *	2.949	1.332	6.529
Home surrounded by buildings	0.004 *	7.008	1.885	26.055
Traffic noise (cars, buses, airplanes, motorcycles) around home † Not at all	<0.001 *			
Slightly	0.010 *	7.264	1.614	32.698
Moderate	0.099	4.111	0.765	22.081
Very	0.640	0.626	0.088	4.459
Extreme	0.028 *	0.059	0.005	0.742
Other noises (sirens, constructions, industry, loading of goods) around home † Not at all	0.023 *			
Slightly	0.004 *	5.03	1.657	15.27
Moderate	0.002 *	8.679	2.203	34.183
Very	0.046 *	6.469	1.038	40.332
Extreme	0.023 *	15.696	1.462	168.53
Natural sounds (singing birds, flowing water, wind in vegetation) around home † Not at all	0.002 *			
Slightly	0.202	1.741	0.744	4.074
Moderate	0.002 *	5.387	1.881	15.431
Very	0.002 *	6.670	1.933	19.04
Extreme	0.517	0.456	0.042	4.911
How loud it around your home † Not at all	<0.001 *			
Slightly	0.018 *	0.122	0.021	0.698
Moderate	0.219	0.309	0.048	2.006
Very	0.157	5.217	0.529	51.422
Extreme	0.012 *	26.666	2.028	350.584
Constant	<0.001 *	0.003		

Dependent variable: cases vs. controls; * represents *p* < 0.05. † Reference.

## Data Availability

Data are available upon request from the corresponding author.
